# Association of NT-proBNP/LVEF with angiographic coronary disease burden in acute myocardial infarction: a retrospective study with machine learning analyses

**DOI:** 10.3389/fcvm.2026.1964997

**Published:** 2026-10-08

**Authors:** Zhongxin Li, Jiantong Hou, Linqing Li, Xianzhao Zhang, Qingrong Ji

**Affiliations:** Department of Cardiology, Linyi People’s Hospital Affiliated to Shandong Second Medical University, Linyi, Shandong, China

**Keywords:** acute myocardial infarction, Gensini score, left ventricular ejection fraction, machine learning, NT-proBNP, severe coronary artery disease, shap, XGBoost

## Abstract

**Background:**

N-terminal pro-B-type natriuretic peptide (NT-proBNP) and left ventricular ejection fraction (LVEF) reflect complementary dimensions of ventricular stress and systolic function. Whether the NT-proBNP/LVEF ratio is consistently associated with angiographic coronary disease burden in patients with acute myocardial infarction (AMI), and whether its predictive contribution is robust across conventional and machine-learning approaches, remains uncertain.

**Methods:**

This single-center retrospective study included 375 patients with AMI who underwent coronary angiography. The continuous Gensini score was the primary outcome, while clinically anchored anatomical outcomes, including multivessel disease and high-burden coronary anatomy, served as sensitivity outcomes. Associations between log-transformed NT-proBNP/LVEF and Gensini score were assessed using multivariable median regression. Direct discrimination of NT-proBNP/LVEF versus NT-proBNP alone was evaluated using the area under the receiver operating characteristic curve (AUC) and paired DeLong testing; the incremental information provided by adding LVEF to NT-proBNP was additionally assessed using continuous net reclassification improvement (NRI) and integrated discrimination improvement (IDI). Six multivariable models—logistic regression, least absolute shrinkage and selection operator (LASSO) logistic regression, random forest, support vector machine (SVM), extreme gradient boosting (XGBoost), and CatBoost—were internally validated using leakage-controlled repeated fivefold cross-validation with five repeats. Calibration, decision curve analysis, and Shapley additive explanations (SHAP)-based model interpretation were also evaluated.

**Results:**

Higher NT-proBNP/LVEF was consistently associated with greater angiographic coronary disease burden. In the fully adjusted model, each doubling of NT-proBNP/LVEF was associated with a 12.20-point increase in median Gensini score (95% confidence interval [CI], 9.33–15.07; *P* < 0.001). Comparable associations were observed for high-burden anatomy (odds ratio [OR] per doubling, 1.69; 95% CI, 1.44–1.97) and multivessel disease (OR per doubling, 1.58; 95% CI, 1.36–1.84). For the secondary high-Gensini-burden outcome, NT-proBNP/LVEF and NT-proBNP showed comparable discrimination (AUC, 0.783 vs. 0.780; ΔAUC = 0.004; *P* = 0.362). Adding LVEF to NT-proBNP did not show clear incremental improvement by NRI or IDI. Among the six multivariable models, SVM yielded the numerically highest AUC (0.763; 95% CI, 0.715–0.811), but no pairwise AUC difference remained significant after correction for multiple comparisons. XGBoost and CatBoost showed highly concordant SHAP importance rankings (Spearman *ρ* = 0.978), with NT-proBNP/LVEF ranked first in both models.

**Conclusions:**

Higher NT-proBNP/LVEF was consistently associated with greater angiographic coronary disease burden across the continuous Gensini score and clinically anchored anatomical outcomes in patients with AMI. The ratio provides a parsimonious representation of ventricular stress and systolic function and showed stable predictive importance across the two boosting algorithms examined. Its discriminative performance was comparable, rather than superior, to that of NT-proBNP alone, supporting its potential role as a readily available adjunctive marker of coronary disease burden rather than a replacement for established clinical assessment.

## 1 Introduction

Acute myocardial infarction (AMI) remains a major cause of cardiovascular morbidity and mortality, yet affected patients show substantial heterogeneity in coronary anatomy, extent of myocardial injury, ventricular function, and subsequent clinical course (1, 2). Coronary angiography provides direct characterization of the underlying coronary lesions, and angiographic disease burden is closely related to clinical risk and therapeutic decision-making (1). Accordingly, routinely available markers that reflect this anatomical burden may provide complementary information during the early assessment of patients with AMI.

N-terminal pro-B-type natriuretic peptide (NT-proBNP) and left ventricular ejection fraction (LVEF) characterize distinct but complementary aspects of the cardiovascular response to AMI. NT-proBNP is released in response to increased myocardial wall stress and is influenced by ventricular filling pressure, neurohumoral activation, myocardial injury, and renal function; natriuretic peptides also provide established prognostic information across acute coronary syndromes (3–5). LVEF, by contrast, remains one of the most widely used measures of left ventricular systolic performance after myocardial infarction, although it is an incomplete marker when used in isolation (6). In clinical practice, marked NT-proBNP elevation and impaired LVEF often coexist, but their changes are not necessarily parallel. Considering the two measures jointly may therefore capture a broader spectrum of cardiac stress and functional impairment than either measure alone.

The NT-proBNP/LVEF ratio offers a simple way to integrate these routinely obtained measurements. A higher ratio may reflect the concurrence of increased ventricular stress and impaired systolic performance, a phenotype that could accompany more extensive myocardial ischemia and greater coronary disease burden. The Gensini score provides a quantitative measure of angiographic disease by incorporating both the severity of luminal stenosis and the anatomical importance of the affected coronary segments (7). Previous studies have linked NT-proBNP to angiographic disease severity, extent of coronary artery disease, and coronary complexity (8–10), but the association between NT-proBNP/LVEF and angiographic coronary disease burden has not been well characterized.

An important methodological consideration is how coronary disease burden is defined. Dichotomizing an angiographic score at a sample-derived threshold may facilitate prediction analyses, but it discards information from the underlying continuous distribution and may yield conclusions that depend on the selected cutoff. We therefore used the continuous Gensini score as the primary measure of disease burden and complemented it with clinically recognizable anatomical phenotypes, including multivessel disease and significant left main disease. This approach enabled assessment of the NT-proBNP/LVEF association across both quantitative and clinically anchored anatomical definitions.

A second question is whether combining NT-proBNP and LVEF into a ratio contributes information beyond NT-proBNP or merely repackages its predictive signal. Ratio variables can impose restrictive relationships between component effects and therefore warrant direct comparison with models containing the components separately. Rather than presuming superiority of the ratio, we directly compared its discrimination with that of NT-proBNP alone and examined whether modeling NT-proBNP and LVEF as separate terms improved model fit. This distinction is clinically relevant because a simple composite marker is useful only when its interpretability and parsimony are supported by the data.

Machine-learning methods provide an additional means of capturing nonlinear relationships and interactions among routinely collected clinical variables. However, greater algorithmic complexity does not necessarily translate into better performance than conventional regression in clinical prediction studies, particularly in modest datasets (11, 12). Their value should therefore not be judged solely by the algorithm with the highest observed discrimination. Reproducibility across resampling procedures, calibration, clinical net benefit, and stability of feature importance may be equally informative. We therefore compared conventional logistic regression with least absolute shrinkage and selection operator (LASSO) logistic regression, random forest, support vector machine (SVM), extreme gradient boosting (XGBoost), and CatBoost within a common repeated cross-validation framework and assessed concordance in model interpretation across the two boosting algorithms.

Accordingly, this study aimed to determine whether NT-proBNP/LVEF is consistently associated with angiographic coronary disease burden in patients with AMI, using the continuous Gensini score as the primary outcome and clinically anchored anatomical outcomes in sensitivity analyses. We further assessed the incremental information provided by the ratio relative to NT-proBNP alone and explored the stability of its predictive contribution across conventional and machine-learning models. The multivariable prediction models were intended for exploratory, internally validated comparison as adjuncts to early clinical assessment rather than as stand-alone tools for immediate clinical implementation.

## 2 Methods

### 2.1 Study population

This single-center retrospective study included patients with AMI admitted to the Department of Cardiology at Linyi People's Hospital who underwent coronary angiography between January 8 and May 28, 2026. AMI was diagnosed on the basis of clinical presentation, electrocardiographic findings, dynamic changes in myocardial injury biomarkers, and coronary angiographic findings, consistent with contemporary definitions and acute coronary syndrome guidance (1, 2). The study was registered internally at Linyi People's Hospital; a publicly accessible study protocol was not available. The observational component was reported in accordance with the STROBE statement, and the exploratory prediction-model analyses were reported with reference to TRIPOD + AI (13, 14).

The source dataset initially contained 379 hospitalization records. After manual verification of hospital identifiers and clinical information, four duplicate records were removed, leaving 375 unique patients in the final analytical cohort. Eligible patients were aged ≥18 years, had a confirmed diagnosis of AMI, underwent coronary angiography during the index hospitalization, and had available NT-proBNP, LVEF, and Gensini score measurements. The final cohort included 161 patients with ST-segment elevation myocardial infarction (STEMI) and 214 with non-ST-segment elevation myocardial infarction (NSTEMI). The study flow is shown in Figure 1. The study size was determined by the number of eligible unique AMI patients in the source dataset during the study period; no formal *a priori* sample-size calculation was performed. The modeling outcome occurred in 204 patients, with 13 prespecified predictors considered in the multivariable models; the available cohort was therefore used for exploratory internal validation rather than definitive model deployment. This reporting approach is consistent with guidance that prediction-model sample size depends on the available outcome information, number of predictor parameters, and anticipated model performance rather than a simple events-per-variable rule alone (15).

**Figure 1:**
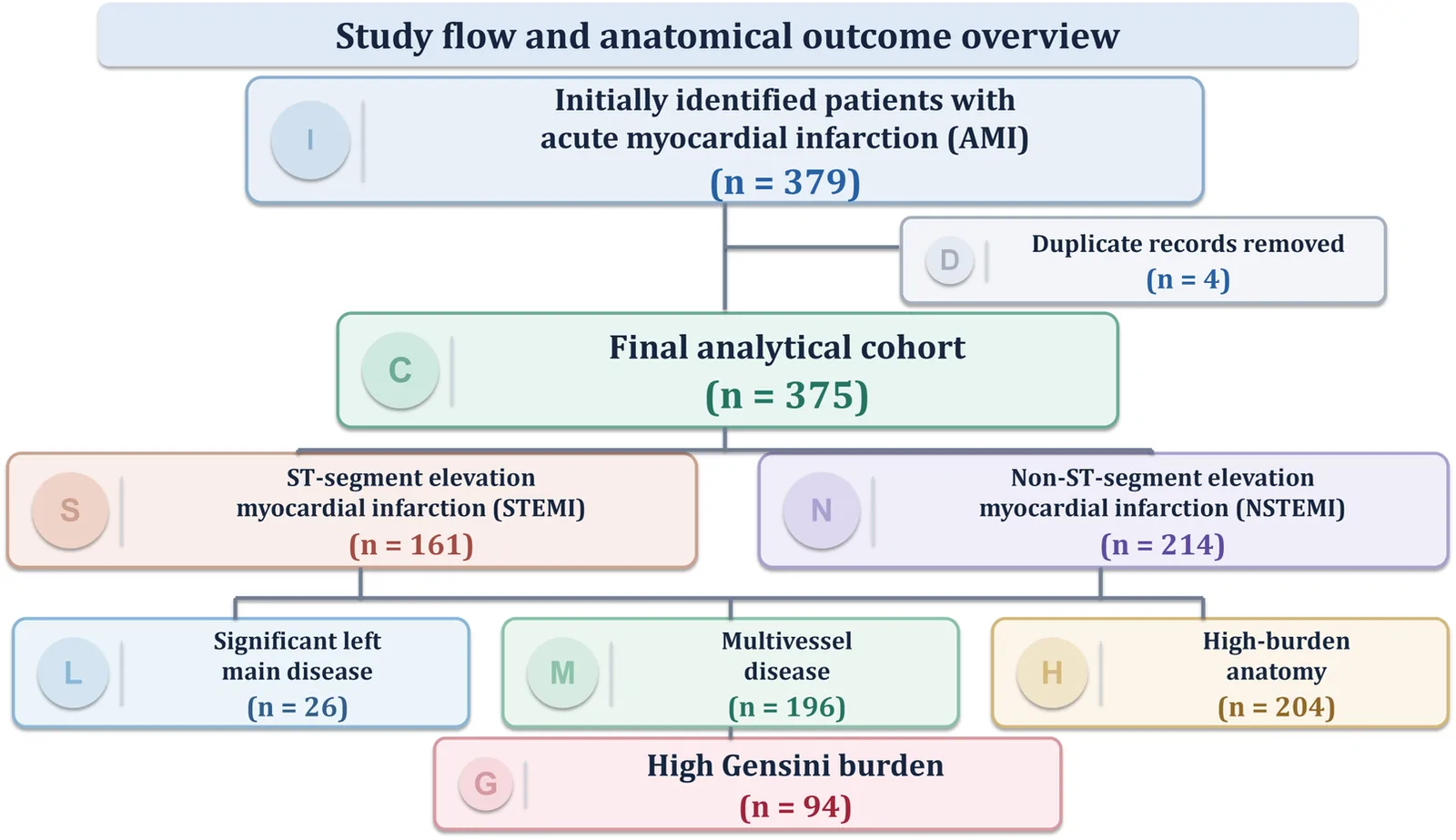
Study flow and overview of angiographic coronary disease burden. The source dataset comprised 379 hospitalization records for acute myocardial infarction (AMI). After manual verification and removal of four duplicate records, 375 unique patients were included in the final analytical cohort, including 161 patients with ST-segment elevation myocardial infarction (STEMI) and 214 with non-ST-segment elevation myocardial infarction (NSTEMI). The figure also summarizes the distribution of clinically anchored angiographic outcomes, including significant left main disease, multivessel disease, high-burden coronary anatomy, and high Gensini burden. High-burden anatomy was defined as significant left main disease (≥50% stenosis) or multivessel disease (≥70% stenosis in at least two major epicardial coronary arteries). High Gensini burden was defined as a Gensini score above the cohort-specific 75th percentile (>95.5). AMI, acute myocardial infarction; STEMI, ST-segment elevation myocardial infarction; NSTEMI, non-ST-segment elevation myocardial infarction.

### 2.2 Data collection and exposure definition

Clinical, laboratory, echocardiographic, and angiographic data were retrospectively extracted from the hospital electronic medical record system. Clinical variables included age, sex, body mass index (BMI), hypertension, and AMI type. Laboratory variables included glucose, blood cell indices, lipid parameters including low-density lipoprotein cholesterol (LDL-C), lipoprotein(a), C-reactive protein (CRP), fibrinogen, D-dimer, troponin T, NT-proBNP, renal function indices, and other routinely measured biochemical parameters. Echocardiographic variables included LVEF and left ventricular end-diastolic diameter (LVEDD).

The first available NT-proBNP measurement during the index hospitalization and the corresponding LVEF measured by transthoracic echocardiography were used for analysis. All NT-proBNP measurements and echocardiographic LVEF assessments used in the present analysis were obtained before coronary angiography as part of the initial in-hospital evaluation. Although the exact time intervals from admission or symptom onset to each examination were not uniformly available for retrospective extraction, the sequence of assessment was confirmed: NT-proBNP measurement and echocardiographic LVEF assessment preceded coronary angiography in all included patients. LVEF was expressed as a percentage, and NT-proBNP/LVEF was calculated as NT-proBNP divided by LVEF. Because both NT-proBNP and NT-proBNP/LVEF were right-skewed, logarithmic transformation was applied where appropriate in regression and predictive modeling.

The principal exposure was log-transformed NT-proBNP/LVEF. To facilitate clinical interpretation, regression effects were additionally expressed per doubling of NT-proBNP/LVEF. For the median regression models, coefficients on the natural-log scale were multiplied by ln(2); for binary outcomes, the corresponding log-odds coefficients were multiplied by ln(2) and exponentiated.

### 2.3 Coronary angiography and assessment of coronary disease burden

All patients underwent coronary angiography during the index hospitalization. Angiographic coronary disease burden was quantified using the Gensini score (7). Briefly, each stenotic lesion was assigned a severity score according to the degree of luminal narrowing and then multiplied by a weighting factor reflecting the anatomical importance of the involved coronary segment. Higher Gensini scores indicated greater angiographic disease burden. Complete occlusion was recorded as 100% stenosis, whereas 99% stenosis denoted subtotal occlusion. Each coronary angiographic procedure was performed by at least two experienced interventional cardiologists. Angiographic findings, including lesion location and stenosis severity, were jointly assessed during the procedure and documented in the formal procedural report. The Gensini score was subsequently calculated from these confirmed angiographic findings according to the standard scoring algorithm. Because the angiographic findings represented a consensus procedural assessment rather than two independent *post hoc* ratings, inter-observer ICC was not applicable.

In addition to the Gensini score, clinically anchored anatomical outcomes were derived from the angiographic records. Significant left main disease was defined as ≥50% stenosis of the left main coronary artery. Multivessel disease was defined as ≥70% stenosis in at least two of the three major epicardial coronary arteries: the left anterior descending, left circumflex, and right coronary arteries. High-burden anatomy was defined as the presence of either significant left main disease or multivessel disease.

### 2.4 Outcome definitions

The primary outcome was angiographic coronary disease burden quantified by the continuous Gensini score. Analyzing the score as a continuous outcome preserved the full range of angiographic information and avoided reliance on a cohort-specific dichotomization threshold.

Significant left main disease was recorded as a clinically relevant anatomical phenotype and constituted one component of the composite high-burden anatomy endpoint. Multivessel disease and composite high-burden anatomy were examined as clinically anchored sensitivity outcomes.

For secondary and exploratory analyses, high Gensini burden was defined as a Gensini score above the 75th percentile of the study cohort. The 75th-percentile cutoff was 95.5, yielding 94 patients with Gensini scores >95.5. This cohort-derived threshold was used for discrimination and incremental-value analyses and was not considered a universal clinical definition of severe coronary artery disease.

### 2.5 Statistical analysis

Continuous variables were summarized as mean ± standard deviation or median (interquartile range), as appropriate for their distributions; categorical variables were summarized as counts and percentages. Between-group comparisons were performed using Welch's *t*-test or the Wilcoxon rank-sum test for continuous variables and the Pearson chi-square test or Fisher's exact test for categorical variables, as appropriate. Standardized mean differences (SMDs) were also reported to quantify between-group differences. To address potential bias inherent to retrospective data collection, uniform eligibility criteria, standardized variable definitions, and consistent outcome definitions were applied across the cohort.

The primary association between NT-proBNP/LVEF and angiographic coronary disease burden was assessed using median regression with the continuous Gensini score as the dependent variable. Because of its right-skewed distribution, NT-proBNP/LVEF was transformed using the natural logarithm. Four prespecified models were fitted sequentially: an unadjusted model; a model adjusted for age and sex; a model additionally adjusted for hypertension and LDL-C; and a fully adjusted model that further included BMI, STEMI presentation, and estimated glomerular filtration rate (eGFR). Regression coefficients were also expressed per doubling of NT-proBNP/LVEF to facilitate clinical interpretation.

Sensitivity analyses for the clinically anchored anatomical outcomes were conducted using multivariable logistic regression for multivessel disease and high-burden anatomy. The same covariate set as in the fully adjusted Gensini model was used. Associations were reported as odds ratios (ORs) with 95% confidence intervals (CIs), including estimates per doubling of NT-proBNP/LVEF.

For secondary analyses, high Gensini burden was defined as a Gensini score above the cohort-specific 75th percentile (>95.5). Discrimination of NT-proBNP/LVEF and NT-proBNP alone was compared using receiver operating characteristic (ROC) curves and the area under the ROC curve (AUC). Differences in AUC were assessed with paired DeLong tests (16), and bootstrap 95% CIs were additionally calculated for ΔAUC. To assess whether adding LVEF provided incremental predictive information beyond NT-proBNP, continuous net reclassification improvement (NRI) and integrated discrimination improvement (IDI) were calculated (17).

Because a ratio imposes a fixed relationship between the coefficients of its two components, an additional formulation analysis compared a restricted model containing log(NT-proBNP/LVEF) with a less constrained model containing log(NT-proBNP) and log(LVEF) as separate terms. Model fit was compared using a likelihood-ratio test.

Prespecified exploratory analyses examined whether the association between NT-proBNP/LVEF and the continuous Gensini score differed across age categories (<65, 65–74, and ≥75 years), together with formal testing of the interaction between age and log-transformed NT-proBNP/LVEF. Other subgroup and sensitivity analyses were considered exploratory. All tests were two-sided, and *P* < 0.05 was considered statistically significant unless otherwise specified.

### 2.6 Missing data handling and internal validation

Missingness was assessed for all candidate predictors before predictive modeling. The highest proportions of missing data were observed for glucose (20.8%), D-dimer (13.6%), CRP (7.7%), fibrinogen (2.9%), and lipoprotein(a) (2.7%). Because missingness for some variables was associated with observed clinical characteristics and outcomes, a missing-completely-at-random mechanism was not assumed.

For descriptive and association analyses, observed data were used, with the effective sample size reported for each fitted model according to variable availability.

For machine-learning analyses, missing predictor values were handled using multiple imputation by chained equations (MICE) within the resampling framework (18). To prevent information leakage, imputation models were estimated using only the training portion of each outer cross-validation split; observations in the corresponding validation fold did not contribute to imputation-model estimation. Outcome status was not included as an imputation predictor. Because NT-proBNP/LVEF is mathematically derived from NT-proBNP and LVEF, the ratio was also excluded from the imputation predictor matrix to avoid deterministic collinearity.

All preprocessing procedures were performed within the corresponding training folds and then applied to the held-out validation data. No information from an outer validation fold was used for predictor preprocessing, imputation, hyperparameter tuning, or model fitting.

Internal validation used repeated fivefold cross-validation with five repeats, yielding 25 outer validation splits. Each patient therefore contributed one out-of-fold prediction per repeat; the five predictions were averaged to obtain a single patient-level out-of-fold probability for final performance evaluation. As a sensitivity analysis, all six predictive models were re-evaluated among patients with complete data for all 13 prespecified predictors using the same repeated fivefold cross-validation framework with five repeats, without multiple imputation. Development and evaluation folds were drawn from the same single-center cohort and therefore used identical eligibility criteria, outcome definitions, predictor definitions, and clinical setting.

### 2.7 Machine-learning model development and evaluation

The primary outcome for predictive modeling was high-burden anatomy. A prespecified predictor set was used to limit data-driven feature selection and ensure comparability across algorithms. The final feature set comprised age, sex, BMI, hypertension, STEMI presentation, glucose, CRP, eGFR, lipoprotein(a), LDL-C, troponin T, LVEDD, and log-transformed NT-proBNP/LVEF. Skewed laboratory variables were log-transformed before modeling where appropriate. Because high-burden anatomy was determined from coronary angiography during the index hospitalization rather than during subsequent follow-up, this component represented a cross-sectional anatomical prediction task and no future prediction horizon was applicable. For algorithms requiring scaled inputs, scaling parameters were estimated within the corresponding training fold and applied unchanged to the held-out validation fold, consistent with leakage-aware model-development practice (12).

Six modeling approaches were compared: conventional logistic regression, LASSO logistic regression, random forest, SVM, XGBoost, and CatBoost (19–21). Logistic regression served as the conventional reference model, whereas the remaining methods represented regularized, kernel-based, bagging, and boosting approaches. Because high-burden anatomy was present in 204 of 375 patients (54.4%), no class-imbalance correction, including over- or undersampling, SMOTE, or class weighting, was applied.

Hyperparameters were optimized within an inner fivefold cross-validation loop nested within each outer training set, ensuring complete separation between tuning and the held-out outer validation observations. Identical outer resampling partitions were used across all six models to enable paired comparisons.

CatBoost was implemented using ordered boosting. Candidate tree depths were tuned within the inner cross-validation procedure, whereas the remaining major hyperparameters were prespecified to limit the complexity of the search. For computational feasibility, CatBoost tuning within each inner fold used the first leakage-free multiply imputed dataset; final predictions for each outer validation fold were generated across all 10 imputed datasets and then averaged. Detailed hyperparameter settings for all algorithms are provided in Supplementary Table S1. All analyses were performed using R version 4.6.0. The analysis environment included mice version 3.19.0, pROC version 1.19.0.1, glmnet version 5.0, randomForest version 4.7.1.2, e1071 version 1.7.17, xgboost version 3.2.1.1, and catboost version 1.2.10. No single algorithm was designated as an implementation model; the six approaches were compared within the same internal-validation framework, and no deployment-ready model object or clinical risk threshold was proposed.

Model discrimination was quantified by ROC AUC with 95% CI and by the area under the precision-recall curve (PR-AUC). Overall probabilistic accuracy was assessed using the Brier score. Pairwise AUC differences among the six models were evaluated using paired DeLong tests (16) and bootstrap estimates of ΔAUC. Because 15 pairwise comparisons were performed, *P* values were adjusted using the Benjamini-Hochberg procedure. Performance measures were interpreted jointly rather than relying on discrimination alone (22).

Calibration was assessed using calibration plots, calibration intercepts, and calibration slopes, with bootstrap 95% CIs (22, 23). An additional NT-proBNP-only model was evaluated under the same repeated cross-validation framework as a clinically interpretable benchmark.

Clinical net benefit was evaluated using decision curve analysis (DCA) (24). Net benefit was examined across threshold probabilities from 0.20 to 0.60 and compared with the treat-all, treat-none, and NT-proBNP-only benchmark strategies. Because formal pairwise testing of net-benefit differences between models was not performed, DCA findings were interpreted descriptively and not as evidence of superiority of any individual model.

Finally, Shapley additive explanations (SHAP) were used to interpret the XGBoost and CatBoost models (25). Global feature importance was summarized by mean absolute SHAP values. Concordance of feature rankings between the two boosting algorithms was assessed using Spearman rank correlation, and overlap among the five highest-ranked features was summarized using the Jaccard index.

## 3 Results

### 3.1 Study population and baseline characteristics

A total of 379 AMI hospitalization records were initially identified. After manual review and removal of four duplicate records, 375 unique patients comprised the final analytical cohort, including 161 patients with STEMI (42.9%) and 214 with NSTEMI (57.1%). According to the prespecified clinically anchored anatomical endpoint, 171 patients had lower-burden anatomy and 204 had high-burden anatomy.

Compared with the lower-burden anatomy group, patients with high-burden anatomy were older (63.3 ± 11.3 vs. 58.2 ± 12.7 years, *P* < 0.001), had lower hemoglobin levels (135.8 ± 18.3 vs. 139.5 ± 16.5, *P* = 0.039), and had higher glucose, lipoprotein(a), and fibrinogen levels but lower triglyceride levels (all *P* < 0.05). eGFR was lower (92.0 ± 18.7 vs. 98.7 ± 15.5, *P* < 0.001), whereas LVEDD was greater (48.3 ± 4.9 vs. 47.2 ± 3.3, *P* = 0.011). No statistically significant between-group differences were observed in sex, BMI, hypertension, STEMI prevalence, LDL-C, high-density lipoprotein cholesterol, troponin T, or LVEF.

NT-proBNP was markedly higher in the high-burden anatomy group than in the lower-burden anatomy group [1,978.0 (872.0–3,437.0) vs. 678.0 (298.4–1,437.5), *P* < 0.001], as was NT-proBNP/LVEF [37.2 (17.0–66.2) vs. 12.0 (5.6–27.6), *P* < 0.001]. The corresponding SMDs were 0.795 and 0.745, respectively. Detailed baseline characteristics are shown in Table 1.

**Table 1:** Baseline characteristics according to angiographic coronary disease burden.

Characteristic	Overall (*N* = 375)	Lower-burden anatomy (*N* = 171)	High-burden anatomy (*N* = 204)	*P*-value	SMD
Age (years)	61.0 ± 12.2	58.2 ± 12.7	63.3 ± 11.3	<0.001	0.431
Male sex	278 (74.1%)	131 (76.6%)	147 (72.1%)	0.316	0.104
BMI (kg/m^2^)	26.1 ± 3.5	26.1 ± 3.3	26.1 ± 3.6	0.947	0.007
Hypertension	207 (55.2%)	96 (56.1%)	111 (54.4%)	0.737	0.035
STEMI	161 (42.9%)	78 (45.6%)	83 (40.7%)	0.337	0.100
White blood cell count (10^9^/L)	8.0 (6.5–10.3)	8.2 (6.7–11.2)	7.9 (6.4–10.1)	0.195	0.175
Hemoglobin (g/L)	137.5 ± 17.6	139.5 ± 16.5	135.8 ± 18.3	0.039	0.216
Platelet count (10^9^/L)	218.0 (189.0–260.8)	225.0 (196.0–263.0)	216.0 (185.0–255.0)	0.096	0.140
Glucose (mmol/L)	6.1 (5.4–7.3)	5.9 (5.4–6.8)	6.3 (5.3–7.7)	0.048	0.239
Total cholesterol (mmol/L)	4.3 (3.7–5.0)	4.5 (3.9–5.2)	4.3 (3.5–5.0)	0.071	0.123
LDL-C (mmol/L)	2.8 (2.2–3.5)	2.9 (2.3–3.5)	2.7 (2.1–3.4)	0.192	0.085
HDL-C (mmol/L)	1.0 (0.9–1.2)	1.0 (0.9–1.2)	1.0 (0.9–1.2)	0.835	0.015
Triglycerides (mmol/L)	1.4 (1.0–2.0)	1.5 (1.1–2.1)	1.3 (1.0–1.9)	0.030	0.291
Lipoprotein(a) (mg/L)	181.8 (91.8–432.2)	144.2 (81.3–364.2)	200.6 (106.5–525.6)	0.005	0.308
C-reactive protein (mg/L)	4.5 (3.1–12.3)	3.4 (3.1–11.0)	4.9 (3.1–13.7)	0.055	0.255
Fibrinogen (g/L)	3.4 (3.0–4.1)	3.4 (3.0–3.9)	3.5 (3.0–4.2)	0.019	0.310
D-dimer (mg/L)	0.3 (0.2–0.6)	0.3 (0.2–0.6)	0.3 (0.2–0.6)	0.709	0.064
Troponin T (ng/mL)	0.9 (0.2–3.7)	1.0 (0.2–4.2)	0.8 (0.2–3.2)	0.897	0.176
NT-proBNP (pg/mL)	1,210.0 (502.2–2,651.5)	678.0 (298.4–1,437.5)	1,978.0 (872.0–3,437.0)	<0.001	0.795
AST (U/L)	49.7 (23.4–150.0)	56.5 (23.2–173.9)	47.0 (23.5–140.8)	0.423	0.080
ALT (U/L)	27.1 (18.2–43.5)	28.3 (18.3–47.5)	26.5 (18.2–41.7)	0.512	0.039
Uric acid (μmol/L)	301.5 (251.4–357.0)	297.0 (253.5–363.3)	303.3 (246.7–353.0)	0.794	0.030
eGFR (mL/min/1.73 m^2^)	95.1 ± 17.7	98.7 ± 15.5	92.0 ± 18.7	<0.001	0.392
LVEF (%)	54.1 ± 5.8	54.7 ± 5.8	53.5 ± 5.8	0.058	0.198
LVEDD (mm)	47.8 ± 4.2	47.2 ± 3.3	48.3 ± 4.9	0.011	0.260
NT-proBNP/LVEF	22.2 (9.3–51.2)	12.0 (5.6–27.6)	37.2 (17.0–66.2)	<0.001	0.745

Data are presented as mean ± SD, median (IQR), or *n* (%), as appropriate. *P*-values were calculated using Welch's *t*-test, Wilcoxon rank-sum test, or Pearson chi-square/Fisher's exact test, as appropriate. SMD denotes standardized mean difference. Analyses used available observations for variables with missing data. High-burden anatomy was defined as significant left main disease (≥50% stenosis) or multivessel disease (≥2 of the LAD, LCX, and RCA with ≥70% stenosis).

BMI, body mass index; STEMI, ST-segment elevation myocardial infarction; LDL-C, low-density lipoprotein cholesterol; HDL-C, high-density lipoprotein cholesterol; NT-proBNP, N-terminal pro-B-type natriuretic peptide; AST, aspartate aminotransferase; ALT, alanine aminotransferase; eGFR, estimated glomerular filtration rate; LVEF, left ventricular ejection fraction; LVEDD, left ventricular end-diastolic diameter; LAD, left anterior descending artery; LCX, left circumflex artery; RCA, right coronary artery.

### 3.2 Association of NT-proBNP/LVEF with continuous Gensini score

With the continuous Gensini score as the primary outcome, median regression demonstrated a strong positive association with log(NT-proBNP/LVEF). The unadjusted regression coefficient was *β* = 17.84 (95% CI, 13.85–21.83; *P* < 0.001). The association remained stable after adjustment for age and sex (*β* = 18.77; 95% CI, 14.76–22.77; *P* < 0.001) and after additional adjustment for hypertension and LDL-C (*β* = 17.26; 95% CI, 13.35–21.17; *P* < 0.001).

In the fully adjusted model, which included age, sex, hypertension, LDL-C, BMI, STEMI presentation, and eGFR, log(NT-proBNP/LVEF) remained associated with a higher Gensini score (*β* = 17.60; 95% CI, 13.46–21.74; *P* < 0.001). On a clinically interpretable scale, each doubling of NT-proBNP/LVEF corresponded to a 12.20-point increase in median Gensini score (95% CI, 9.33–15.07). Effect estimates were directionally consistent across all levels of adjustment (Table 2; Figure 2A). In prespecified age-stratified analyses, the association remained positive in patients aged <65 years (*β* = 13.62; 95% CI, 6.35–20.89), 65–74 years (*β* = 17.04; 95% CI, 8.96–25.12), and ≥75 years (*β* = 21.90; 95% CI, 5.02–38.78), with no evidence of interaction by age (*P* for interaction = 0.344).

**Table 2:** Association of NT-proBNP/LVEF With continuous Gensini score in median regression models.

Model	Adjustment	*N*	β (95% CI)	β per doubling (95% CI)	*P*-value
Model 1	Unadjusted	375	17.84 (13.85–21.83)	12.37 (9.60–15.13)	<0.001
Model 2	Age and sex	375	18.77 (14.76–22.77)	13.01 (10.23–15.78)	<0.001
Model 3	Age, sex, hypertension, and LDL-C	368	17.26 (13.35–21.17)	11.96 (9.25–14.67)	<0.001
Model 4	Age, sex, hypertension, LDL-C, BMI, STEMI, and eGFR	363	17.60 (13.46–21.74)	12.20 (9.33–15.07)	<0.001

Values are regression coefficients (β) with 95% confidence intervals from median regression models. β per doubling represents the estimated difference in median Gensini score associated with a twofold increase in NT-proBNP/LVEF and was calculated as β × ln(2).

BMI, body mass index; CI, confidence interval; eGFR, estimated glomerular filtration rate; LDL-C, low-density lipoprotein cholesterol; LVEF, left ventricular ejection fraction; NT-proBNP, N-terminal pro-B-type natriuretic peptide; STEMI, ST-segment elevation myocardial infarction.

**Figure 2:**
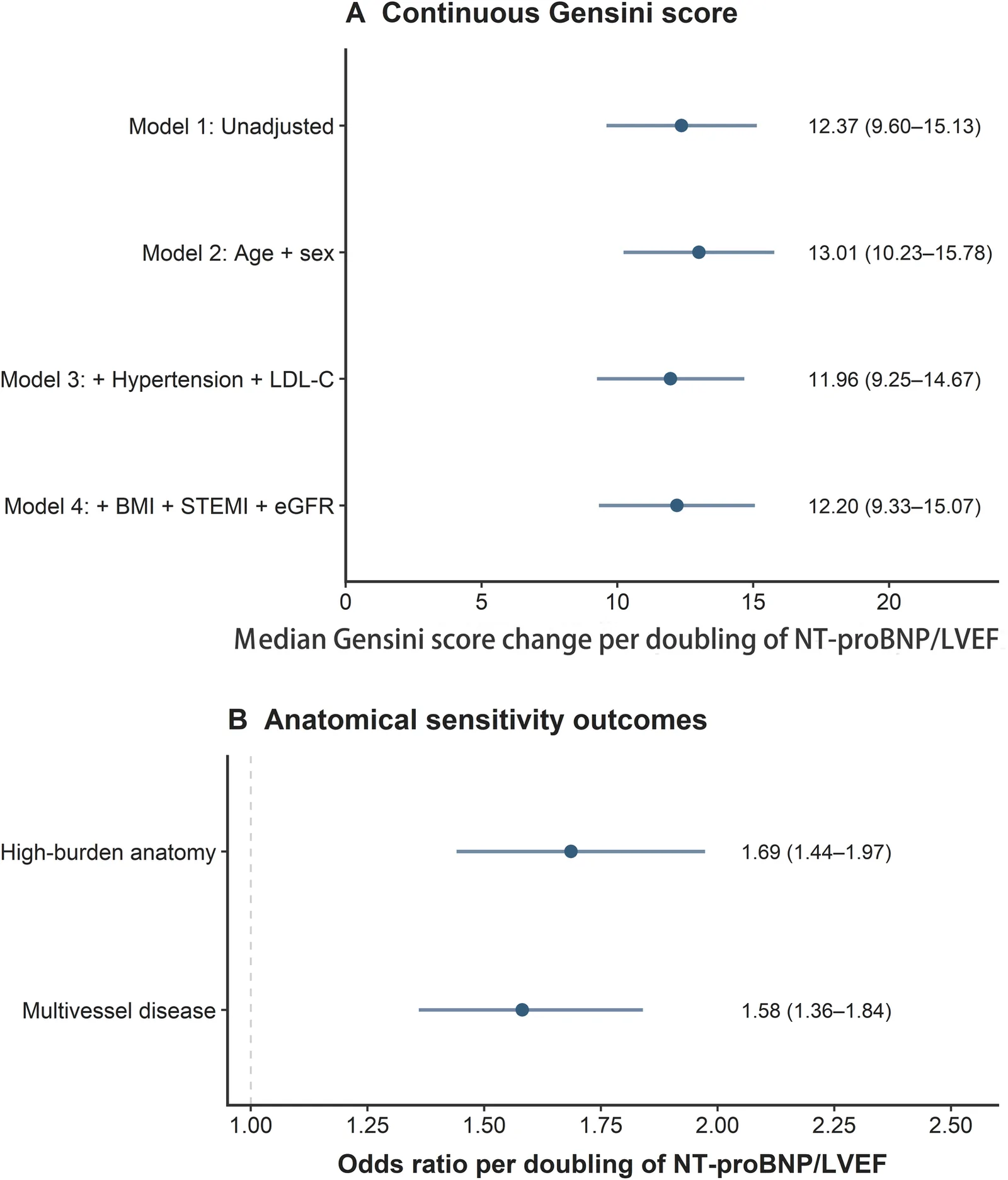
Association of NT-proBNP/LVEF with angiographic coronary disease burden. **(A)** Forest plot showing the association between log-transformed NT-proBNP/LVEF and the continuous Gensini score across sequential median regression models. Models were progressively adjusted for age, sex, hypertension, low-density lipoprotein cholesterol, body mass index, STEMI presentation, and estimated glomerular filtration rate. **(B)** Associations of NT-proBNP/LVEF with clinically anchored anatomical outcomes, including multivessel disease and high-burden anatomy, estimated using multivariable logistic regression with the fully adjusted covariate set. Effect estimates are presented with 95% confidence intervals; effects per doubling of NT-proBNP/LVEF are provided to facilitate clinical interpretation. NT-proBNP, N-terminal pro-B-type natriuretic peptide; LVEF, left ventricular ejection fraction; CI, confidence interval; OR, odds ratio; STEMI, ST-segment elevation myocardial infarction; LDL-C, low-density lipoprotein cholesterol; BMI, body mass index; eGFR, estimated glomerular filtration rate.

### 3.3 Clinically anchored anatomical sensitivity analyses

To assess whether the primary association extended to clinically recognizable coronary phenotypes, multivessel disease and high-burden anatomy were examined as sensitivity outcomes. Among the 375 patients, 196 had multivessel disease, 204 met the definition of high-burden anatomy, and 26 had significant left main disease.

After full adjustment for age, sex, hypertension, LDL-C, BMI, STEMI presentation, and eGFR, higher NT-proBNP/LVEF remained associated with high-burden anatomy (OR = 2.13; 95% CI, 1.69–2.67; *P* < 0.001) and multivessel disease (OR = 1.94; 95% CI, 1.56–2.41; *P* < 0.001). Expressed per doubling, the corresponding ORs were 1.69 (95% CI, 1.44–1.97) and 1.58 (95% CI, 1.36–1.84), respectively (Figure 2B). These results were consistent with the continuous Gensini analysis and were not dependent on a single dichotomization threshold.

### 3.4 Incremental value and model formulation

For the secondary high-Gensini-burden outcome (Gensini score >95.5), NT-proBNP/LVEF and NT-proBNP alone showed similar discrimination. The AUC was 0.783 for NT-proBNP/LVEF and 0.780 for NT-proBNP, corresponding to a small, non-significant difference (ΔAUC = 0.004; 95% CI, −0.004 to 0.011; paired DeLong *P* = 0.362).

In adjusted models, AUCs were 0.778 for NT-proBNP, 0.784 for NT-proBNP/LVEF, and 0.786 for NT-proBNP and LVEF modeled as separate terms. Adding LVEF to NT-proBNP yielded a continuous NRI of 0.306 (95% CI, −0.057 to 0.531) and an IDI of 0.008 (95% CI, approximately −0.0001 to 0.044), neither of which provided clear evidence of incremental discrimination.

When the restricted log(NT-proBNP/LVEF) formulation was compared with the model estimating log(NT-proBNP) and log(LVEF) separately, deviance decreased from 342.648 to 341.773, but the likelihood-ratio test was not significant (statistic = 0.874; 1 degree of freedom; *P* = 0.350). Thus, the more parsimonious ratio formulation showed no clear loss of model fit while maintaining discrimination comparable to that of NT-proBNP alone (Table 3).

**Table 3:** Incremental discrimination and model-formulation analyses of NT-proBNP/LVEF for high gensini burden.

Panel A. Single-marker discrimination and incremental indices			
Analysis	Estimate	95% CI	*P*-value
NT-proBNP alone	AUC 0.780	–	–
NT-proBNP/LVEF	AUC 0.783	–	–
Delta AUC: ratio vs. NT-proBNP	0.004	−0.004 to 0.011	0.362
NRI: adding LVEF to NT-proBNP	0.306	−0.057 to 0.531	–
IDI: adding LVEF to NT-proBNP	0.008	−0.0001 to 0.044	–

Panel B. Adjusted model formulation comparison		
Model	Formulation	AUC
Adjusted NT-proBNP model	log(NT-proBNP)	0.778
Adjusted ratio model	log(NT-proBNP/LVEF)	0.784
Adjusted NT-proBNP + LVEF model	log(NT-proBNP) + log(LVEF)	0.786

Panel C. Likelihood-ratio test of the ratio constraint					
Comparison	Restricted deviance	Free-model deviance	LR statistic	df	*P*-value
Ratio formulation vs. separately estimated NT-proBNP and LVEF terms	342.648	341.773	0.874	1	0.350

The endpoint for this table was the cohort-specific high Gensini burden, defined as a Gensini score >95.5 (75th percentile). The paired DeLong test was used for the AUC comparison between NT-proBNP/LVEF and NT-proBNP. NRI and IDI estimates are presented with 95% confidence intervals. In Panel B, the adjusted models used the same covariate framework; the likelihood-ratio test in Panel C assessed whether estimating NT-proBNP and LVEF as separate terms improved model fit over the ratio formulation. A dash indicates that the corresponding value was not reported for that analysis.

AUC, area under the receiver operating characteristic curve; CI, confidence interval; IDI, integrated discrimination improvement; LVEF, left ventricular ejection fraction; NRI, net reclassification improvement; NT-proBNP, N-terminal pro-B-type natriuretic peptide.

### 3.5 Repeated cross-validated performance of six multivariable models

All six multivariable models were evaluated using the same repeated 5 × 5 outer cross-validation framework and patient-level averaged out-of-fold predictions. Overall discrimination was moderate. SVM yielded the numerically highest AUC at 0.763 (95% CI, 0.715–0.811), together with the highest PR-AUC (0.799) and lowest Brier score (0.199). The AUCs were 0.753 (95% CI, 0.704–0.802) for logistic regression, 0.751 (95% CI, 0.702–0.801) for XGBoost, 0.751 (95% CI, 0.702–0.800) for LASSO logistic regression, 0.740 (95% CI, 0.690–0.790) for CatBoost, and 0.733 (95% CI, 0.682–0.784) for random forest (Table 4; Figure 3).

**Table 4:** Performance of six multivariable models under unified 5 × 5 out-of-fold validation.

Model	AUC (95% CI)	PR-AUC	Brier score	Calibration intercept (95% CI)	Calibration slope (95% CI)
Support vector machine	0.763 (0.715–0.811)	0.799	0.199	0.013 (−0.204–0.229)	1.077 (0.821–1.399)
Logistic regression	0.753 (0.704–0.802)	0.773	0.202	−0.010 (−0.261–0.230)	0.820 (0.622–1.083)
XGBoost	0.751 (0.702–0.801)	0.767	0.202	0.005 (−0.261–0.243)	0.850 (0.647–1.113)
LASSO logistic regression	0.751 (0.702–0.800)	0.770	0.202	−0.004 (−0.213–0.232)	1.055 (0.793–1.375)
CatBoost	0.740 (0.690–0.790)	0.756	0.207	−0.024 (−0.265–0.228)	0.762 (0.573–0.974)
Random forest	0.733 (0.682–0.784)	0.748	0.208	0.007 (−0.224–0.245)	1.093 (0.815–1.423)

Performance was evaluated from patient-level out-of-fold predictions obtained using five repeats of five-fold outer cross-validation (*N* = 375; 204 high-burden anatomy events). High-burden anatomy was defined as significant left main disease (≥50% stenosis) or multivessel disease. No pairwise difference in AUC remained statistically significant after Benjamini–Hochberg correction for multiple comparisons. Calibration intercepts closer to 0 and slopes closer to 1 indicate better calibration. Calibration intercepts and slopes are presented with bootstrap 95% confidence intervals.

AUC, area under the receiver operating characteristic curve; CI, confidence interval; PR-AUC, area under the precision–recall curve; LASSO, least absolute shrinkage and selection operator; SVM, support vector machine; XGBoost, extreme gradient boosting.

**Figure 3:**
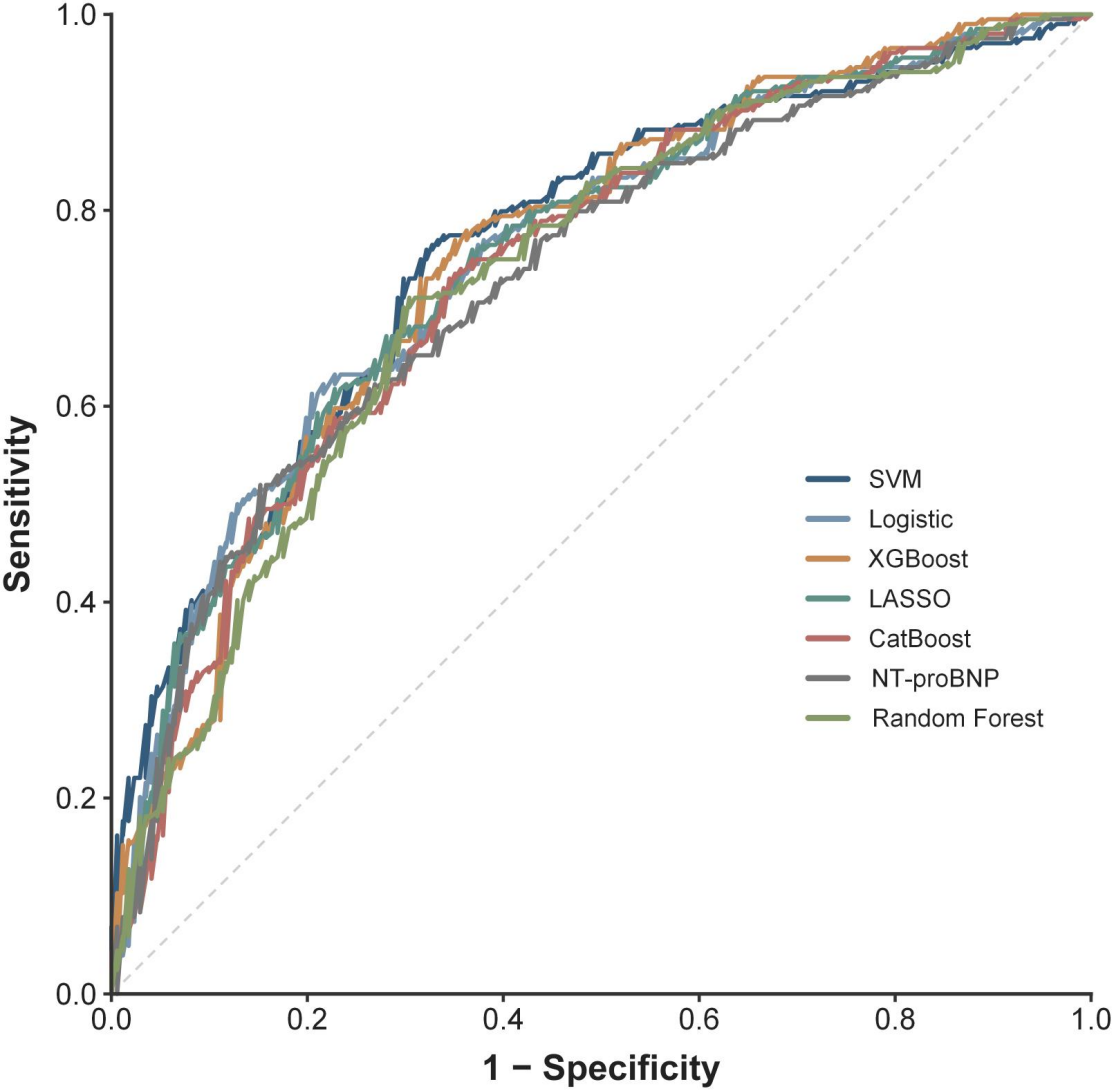
Cross-validated receiver operating characteristic curves for prediction of high-burden coronary anatomy. Receiver operating characteristic (ROC) curves were generated from patient-level averaged out-of-fold predictions obtained using repeated fivefold cross-validation with five repeats. Six multivariable approaches—logistic regression, least absolute shrinkage and selection operator (LASSO) logistic regression, random forest, support vector machine (SVM), extreme gradient boosting (XGBoost), and CatBoost—were evaluated within identical outer resampling partitions. An NT-proBNP-only model was included as a clinically interpretable benchmark. Area under the ROC curve values were calculated from the corresponding out-of-fold predictions. ROC, receiver operating characteristic; AUC, area under the receiver operating characteristic curve; LASSO, least absolute shrinkage and selection operator; SVM, support vector machine; XGBoost, extreme gradient boosting; NT-proBNP, N-terminal pro-B-type natriuretic peptide.

Despite these numerical differences, no pairwise comparison of AUC remained statistically significant after Benjamini-Hochberg correction. For example, the AUC for XGBoost exceeded that for CatBoost by 0.012, but the difference was not significant (DeLong *P* = 0.160; bootstrap 95% CI for ΔAUC, −0.004 to 0.029). Likewise, SVM did not demonstrate a statistically robust advantage over logistic regression, XGBoost, or LASSO. Thus, greater algorithmic complexity did not translate into clear superiority over conventional logistic regression in this cohort. In the complete-case cohort (*n* = 259; 145 patients with high-burden anatomy), model discrimination remained broadly consistent with the primary analysis, with AUCs ranging from 0.737 to 0.769 and differences from the corresponding primary-analysis estimates ranging from −0.015 to +0.018 (Supplementary Table S2).

### 3.6 Calibration and clinical net benefit

Bootstrap calibration analysis showed intercepts close to 0 for all six multivariable models: −0.010 (95% CI, −0.261 to 0.230) for logistic regression, −0.004 (−0.213 to 0.232) for LASSO, 0.007 (−0.224 to 0.245) for random forest, 0.013 (−0.204 to 0.229) for SVM, 0.005 (−0.261 to 0.243) for XGBoost, and −0.024 (−0.265 to 0.228) for CatBoost. Calibration slopes were 0.820 (95% CI, 0.622–1.083), 1.055 (0.793–1.375), 1.093 (0.815–1.423), 1.077 (0.821–1.399), 0.850 (0.647–1.113), and 0.762 (0.573–0.974), respectively. CatBoost was the only model for which the calibration-slope 95% CI excluded 1, suggesting somewhat overly extreme predicted probabilities (Figure 4). With an outcome prevalence of 54.4%, the null Brier score was 0.248; Brier skill scores were 0.186 for logistic regression, 0.184 for LASSO, 0.163 for random forest, 0.198 for SVM, 0.185 for XGBoost, and 0.165 for CatBoost.

**Figure 4:**
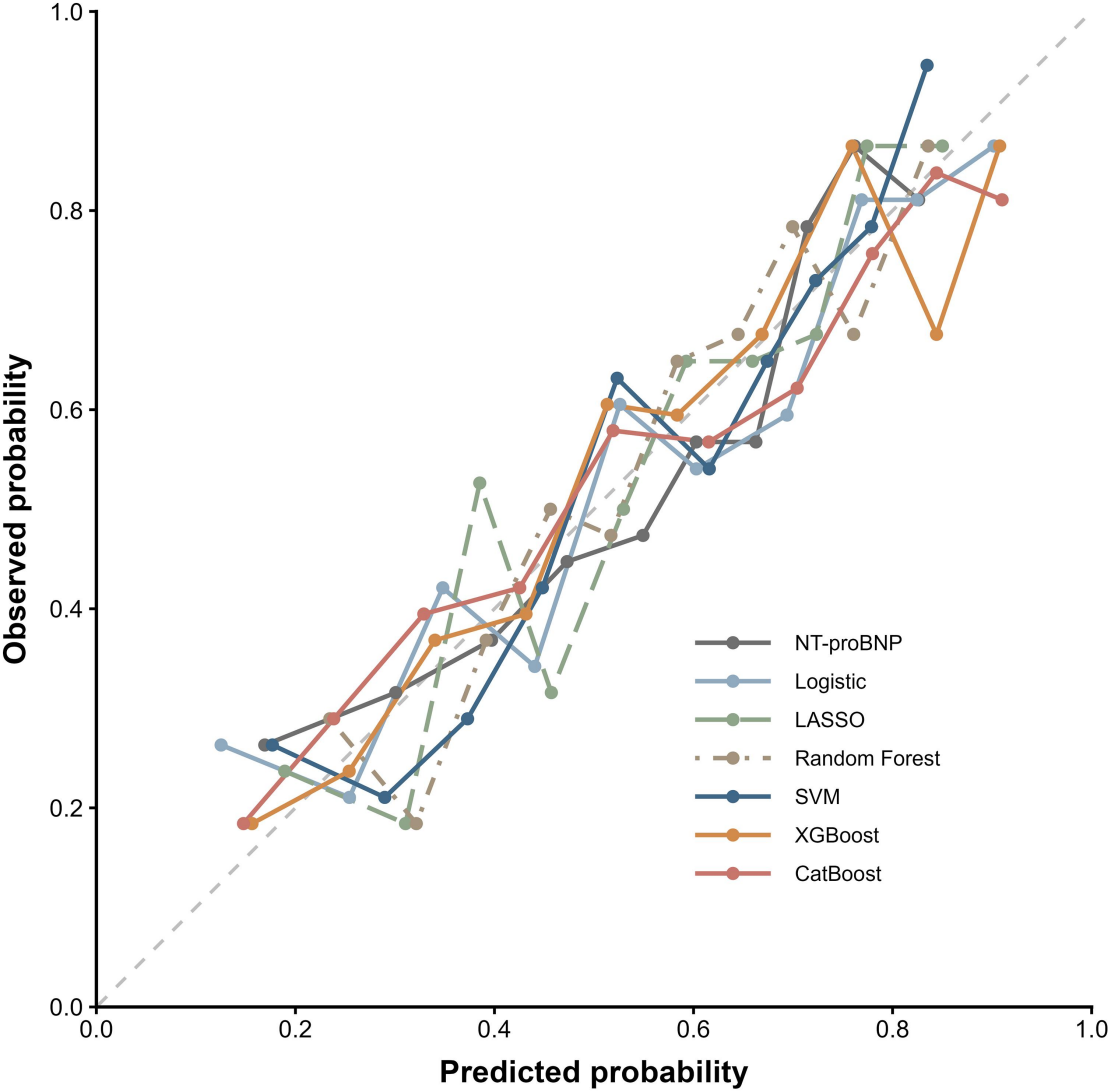
Calibration of models for prediction of high-burden coronary anatomy. Calibration curves compare observed outcome frequencies with predicted probabilities based on patient-level averaged out-of-fold predictions derived from repeated fivefold cross-validation with five repeats. Calibration was evaluated for the six multivariable models and the NT-proBNP-only benchmark. The diagonal reference line represents perfect agreement between predicted and observed probabilities. NT-proBNP, N-terminal pro-B-type natriuretic peptide; LASSO, least absolute shrinkage and selection operator; SVM, support vector machine; XGBoost, extreme gradient boosting.

DCA indicated that several multivariable models provided greater net benefit than the NT-proBNP-only benchmark and the treat-all/treat-none strategies over portions of the prespecified threshold-probability range of 0.20–0.60. Model ranking varied by threshold, and no single model dominated across the entire range. Accordingly, DCA was interpreted as descriptive evidence of potential net benefit rather than proof of superiority of any individual algorithm (Figure 5).

**Figure 5:**
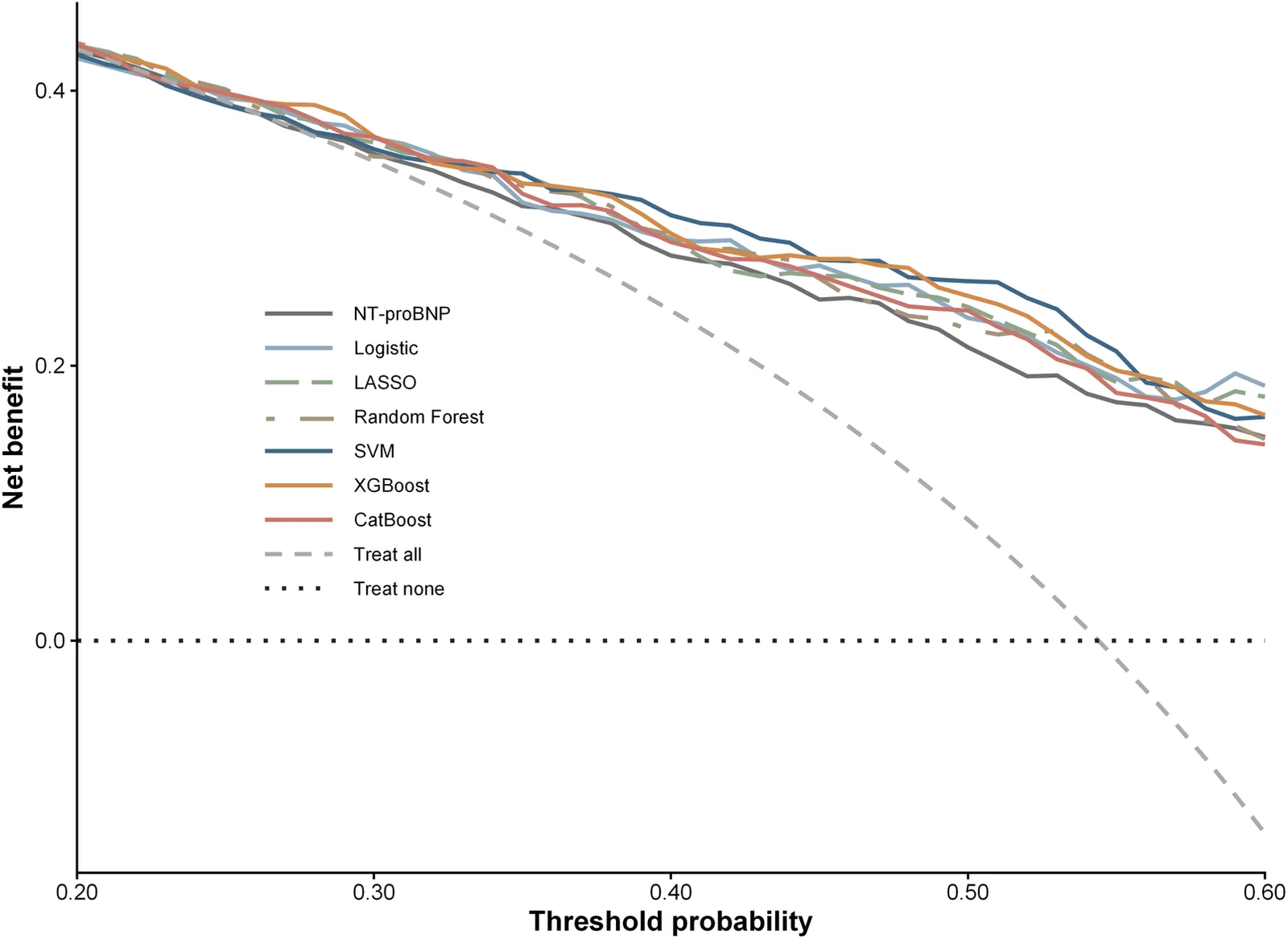
Decision curve analysis of models for prediction of high-burden coronary anatomy. Decision curve analysis was performed using patient-level averaged out-of-fold predicted probabilities derived from repeated fivefold cross-validation with five repeats. Net benefit was evaluated across prespecified threshold probabilities from 0.20 to 0.60. The six multivariable models and the NT-proBNP-only benchmark were compared with the treat-all and treat-none strategies. Model rankings varied across threshold probabilities; therefore, the curves were interpreted descriptively and were not used to establish statistical superiority of any individual algorithm. DCA, decision curve analysis; NT-proBNP, N-terminal pro-B-type natriuretic peptide.

### 3.7 Concordance of XGBoost and CatBoost SHAP interpretation

SHAP analysis of XGBoost and CatBoost was used to determine whether the two boosting algorithms identified similar sources of predictive information. Within the prespecified ratio-based feature set, log(NT-proBNP/LVEF) ranked first by mean absolute SHAP value in both models. Troponin T, glucose, eGFR, and LVEDD were also among the most influential features (Figure 6).

**Figure 6:**
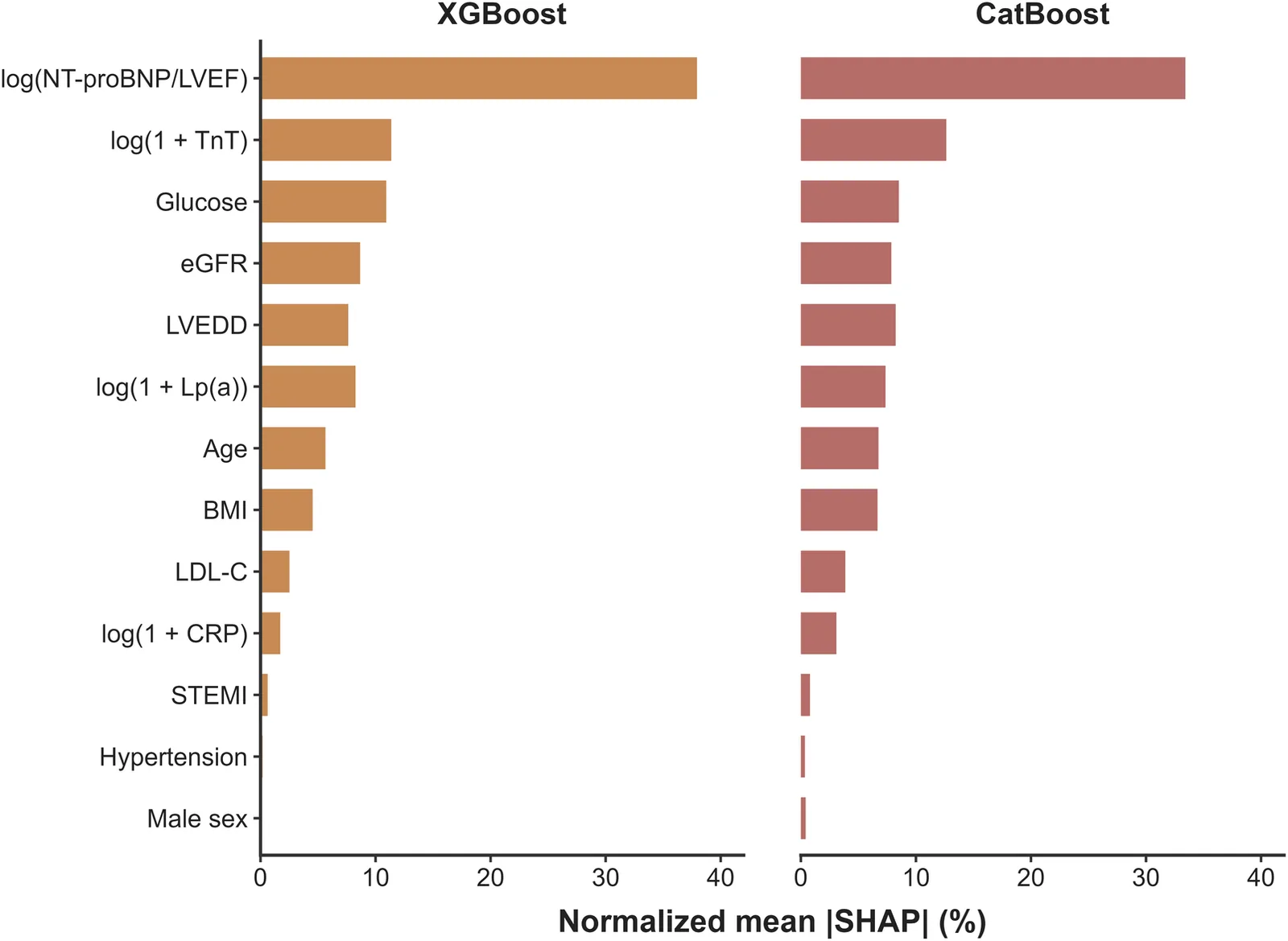
SHAP-based feature importance for XGBoost and CatBoost models. Global feature importance was evaluated using Shapley additive explanations (SHAP) for the XGBoost and CatBoost models developed using the prespecified ratio-based predictor set. Features are displayed in a common order to facilitate cross-model comparison, while importance within each model was quantified using mean absolute SHAP values. Mean absolute SHAP values were normalized within each model to sum to 100%, thereby representing the relative contribution of each feature to model predictions. Log-transformed NT-proBNP/LVEF ranked first in both algorithms. Feature-importance rankings were highly concordant between XGBoost and CatBoost (Spearman *ρ* = 0.978), with four of the five highest-ranked features shared between the two models (Jaccard index = 0.667). SHAP importance reflects predictive contribution within the fitted models and should not be interpreted as evidence of causality or as proof of incremental predictive superiority of the ratio over NT-proBNP alone. SHAP, Shapley additive explanations; XGBoost, extreme gradient boosting; NT-proBNP, N-terminal pro-B-type natriuretic peptide; LVEF, left ventricular ejection fraction; TnT, troponin T; eGFR, estimated glomerular filtration rate; LVEDD, left ventricular end-diastolic diameter; Lp**(a)**, lipoprotein**(a)**; BMI, body mass index; LDL-C, low-density lipoprotein cholesterol; CRP, C-reactive protein; STEMI, ST-segment elevation myocardial infarction.

Feature-importance rankings were highly concordant between XGBoost and CatBoost (Spearman *ρ* = 0.978, *P* < 0.001). Four of the five highest-ranked features overlapped, corresponding to a Jaccard index of 0.667. This cross-algorithm agreement indicates that the principal sources of predictive information were stable across the two boosting approaches. Importantly, the first-place ranking of NT-proBNP/LVEF applies to the prespecified ratio-based feature set and should not be interpreted as evidence that the ratio is superior to NT-proBNP alone; the direct incremental-value analyses did not demonstrate such superiority.

## 4 Discussion

The central finding of this study is that NT-proBNP/LVEF showed a stable, internally consistent association with angiographic coronary disease burden in patients with AMI. Rather than relying on a single dichotomous threshold, we first analyzed the Gensini score as a continuous outcome and then examined multivessel disease and high-burden anatomy as clinically anchored sensitivity outcomes. Across these complementary definitions, higher NT-proBNP/LVEF consistently identified patients with greater coronary disease burden. The association persisted after adjustment for age, sex, hypertension, LDL-C, BMI, STEMI presentation, and eGFR; on a clinically intuitive scale, each doubling of the ratio corresponded to an approximately 12-point increase in median Gensini score. This consistency across quantitative and anatomical outcomes extends the interpretation of NT-proBNP/LVEF beyond a cohort-specific high-risk cutoff and supports its relevance as an integrated cardiac phenotype related to coronary anatomical burden.

This association is also biologically plausible. NT-proBNP reflects ventricular wall stress, filling pressure, and neurohumoral activation, whereas LVEF reflects global left ventricular systolic performance (3–5). These measures are related but not interchangeable: pronounced NT-proBNP elevation may occur despite relatively preserved LVEF, while substantial systolic dysfunction can be observed across a wide range of natriuretic peptide concentrations. The NT-proBNP/LVEF ratio therefore captures two complementary dimensions of cardiac status in a single measure, with higher values potentially reflecting the coexistence of greater ventricular stress and poorer systolic function. Previous studies have separately linked NT-proBNP to angiographic disease severity and coronary complexity (8–10), whereas systematic evaluation of its ratio with LVEF against both quantitative and clinically anchored coronary anatomy remains limited.

A particularly informative aspect of the present analysis is that the observed association was not confined to the Gensini score. Although the Gensini system provides a detailed quantitative representation of stenosis severity and lesion location, multivessel disease and significant left main disease are more directly recognizable anatomical phenotypes in clinical practice and are closely linked to risk assessment and revascularization decisions. NT-proBNP/LVEF remained associated with multivessel disease and the composite high-burden anatomy endpoint after full covariate adjustment. The persistence of the association when moving from a weighted continuous score to clinically interpretable anatomical phenotypes strengthens the internal coherence of the findings.

The direct comparison of ratio and component formulations yielded another useful finding. A common concern with ratio variables is that combining two measurements into a single quantity may discard information or impose a restrictive relationship between component effects. We therefore compared log(NT-proBNP/LVEF) with a less constrained model in which log(NT-proBNP) and log(LVEF) were estimated separately. The separate-component model did not significantly improve model fit. In this cohort, therefore, the ratio provided a more parsimonious representation without an evident penalty in fit, offering a statistical rationale for its use in addition to its physiological interpretability.

wImportantly, parsimony does not imply superior discrimination. NT-proBNP/LVEF and NT-proBNP alone yielded nearly identical AUCs for high Gensini burden, whereas adding LVEF to NT-proBNP did not show a clear incremental gain by NRI or IDI. Rather than undermining the value of the ratio, this finding helps define its role more precisely. NT-proBNP is already highly informative, and a simple transformation would not necessarily be expected to produce a large additional increase in discrimination. The potential value of NT-proBNP/LVEF may therefore lie less in improving AUC and more in integrating two complementary aspects of cardiac stress and function into a single clinically interpretable phenotype.

The machine-learning analysis provided a complementary perspective. Logistic regression, LASSO, random forest, SVM, XGBoost, and CatBoost were evaluated within the same repeated 5 × 5 cross-validation framework, thereby minimizing the influence of a favorable single train-test split. Their AUCs clustered within a relatively narrow range. Although SVM yielded the numerically highest AUC, no model retained a significant advantage after correction for multiple comparisons. These findings indicate that, in this dataset, increasing algorithmic complexity did not automatically translate into better discrimination.

This observation is methodologically relevant. Clinical machine-learning studies often emphasize the model with the highest observed AUC, even when absolute differences are small and potentially sensitive to sampling variation. Systematic evidence has shown that machine-learning algorithms do not automatically outperform logistic regression in clinical prediction modeling (11), and careful preprocessing, tuning, and validation are essential for fair comparisons (12). In a dataset of this size and dimensionality, a conventional logistic model may remain highly competitive, while the additional flexibility of nonlinear or ensemble methods offers limited incremental gain. Our results therefore underscore the importance of a rigorous, shared validation framework when comparing algorithms, rather than selecting a “best” model from a single split.

Calibration and DCA reinforced this interpretation. Calibration should be assessed alongside discrimination when prediction models are compared (22, 23). Most models showed reasonably centered calibration, although CatBoost had a calibration slope below 1, consistent with somewhat overly extreme predicted probabilities. Across the prespecified threshold range, several multivariable models achieved greater net benefit than default or NT-proBNP-only strategies, but the leading model varied by threshold. Decision-curve findings were therefore interpreted descriptively (24). Thus, discrimination, calibration, and net benefit converged on the same conclusion: no single algorithm was consistently dominant.

The SHAP analyses added a different and potentially more informative layer. SHAP provides a theoretically grounded approach to attributing tree-model predictions to individual features (25). XGBoost and CatBoost produced highly concordant feature-importance rankings, with a Spearman correlation of approximately 0.98. NT-proBNP/LVEF ranked first in both models, while troponin T, glucose, eGFR, and LVEDD repeatedly appeared among the most influential features. Despite differences in boosting implementation, the two algorithms identified a strikingly similar distribution of predictive information. This cross-algorithm stability supports the robustness of the ratio's prominence within the prespecified feature set.

SHAP importance nevertheless reflects contribution to model prediction rather than causality or biomarker superiority. The most direct comparison between NT-proBNP/LVEF and NT-proBNP remains the discrimination and reclassification analysis, which indicated comparable rather than superior performance. Taken together, these findings provide a coherent interpretation: NT-proBNP/LVEF carries substantial information within multivariable models and is consistently associated with coronary disease burden, while its principal advantage lies in integration and interpretability rather than a proven increase in discrimination over NT-proBNP alone.

From a clinical perspective, the contribution of this work is not to replace coronary angiography or established risk assessment, but to derive additional information from measurements already obtained routinely in patients with AMI. Marked NT-proBNP elevation in the setting of reduced LVEF produces a high NT-proBNP/LVEF value; in our cohort, this pattern was associated not only with higher Gensini scores but also with more complex coronary anatomy. Because the ratio requires no additional testing, it may offer a convenient adjunct to early clinical assessment when interpreted alongside symptoms, electrocardiography, troponin, renal function, echocardiography, and angiographic findings.

## 5 Limitations

Several limitations merit consideration. First, the single-center retrospective design and moderate sample size may limit generalizability and leave residual confounding despite multivariable adjustment. Several potentially relevant covariates, including diabetes, smoking, prior coronary disease or PCI, Killip class, and pre-admission medication use, were not consistently available as reliable structured fields across the cohort. Second, although NT-proBNP and LVEF were obtained before coronary angiography in all patients, both are acute-phase cardiac measures and may partly reflect the severity and hemodynamic consequences of the index infarction rather than coronary anatomical burden alone. Third, although repeated cross-validation was used to reduce optimism in model evaluation, the predictive models have not yet been externally validated. Finally, NT-proBNP/LVEF is a composite measure, and similar ratio values may arise from different combinations of NT-proBNP elevation and LVEF reduction. Multicenter studies are therefore needed to confirm generalizability and to clarify whether distinct component patterns represent clinically meaningful phenotypes. Formal fairness analyses of predictive performance across sociodemographic subgroups were not performed and should be addressed in larger, more diverse external cohorts.

## 6 Conclusion

In patients with AMI, higher NT-proBNP/LVEF was consistently associated with greater angiographic coronary disease burden across the continuous Gensini score and clinically anchored anatomical outcomes. The ratio provided a parsimonious representation of combined information on ventricular stress and systolic function without clear evidence of meaningful information loss compared with modeling NT-proBNP and LVEF separately. Although its discrimination was comparable rather than superior to that of NT-proBNP alone, NT-proBNP/LVEF showed stable feature importance across the two boosting algorithms examined and may serve as a readily available adjunctive marker of coronary disease burden in AMI. External validation is required before broader clinical application.

## Data Availability

The original contributions presented in the study are included in the article/Supplementary Material, further inquiries can be directed to the corresponding author.
