# Supplementary material for: Association of NT-proBNP/LVEF with angiographic coronary disease burden in acute myocardial infarction: a retrospective study with machine learning analyses
Source: Front Cardiovasc Med. 2026 Oct 8;13:1964997. doi: 10.3389/fcvm.2026.1964997 (PMC13687254; doi:10.3389/fcvm.2026.1964997)
Supplement: Supplementary file 1 [file Table1.docx]

**Supplementary Table S1. Model tuning strategy, hyperparameter settings, and software environment**

| **Model** | **Hyperparameters evaluated** | **Selection across the 25 outer folds / fixed settings** |
| --- | --- | --- |
| **Logistic regression** | None | Conventional multivariable logistic regression; no hyperparameter tuning. |
| **LASSO logistic regression** | Regularization parameter λ selected within the inner 5-fold cross-validation loop. | Across the 25 outer folds, median selected λ = 0.01318 (IQR, 0.00916–0.01896; range, 0.00072–0.11679). |
| **Random forest** | mtry = {3, 6}; min.node.size = {5, 15}. | Selected combinations across 25 outer folds: 3/5 (n=8), 3/15 (n=7), 6/5 (n=7), and 6/15 (n=3). |
| **Support vector machine** | cost = {0.5, 2}; gamma = {0.03, 0.08}. | Selected cost/gamma combinations: 0.5/0.03 (n=8), 0.5/0.08 (n=2), 2/0.03 (n=14), and 2/0.08 (n=1). |
| **XGBoost** | max_depth = {2, 3}; eta = {0.03, 0.10}. | Selected max_depth/eta combinations: 2/0.03 (n=11), 2/0.10 (n=10), and 3/0.10 (n=4); 3/0.03 was not selected. |
| **CatBoost** | depth = {4, 6}. | Depth 4 was selected in 16 folds and depth 6 in 9 folds. Fixed settings: Ordered boosting; SymmetricTree; 200 iterations; learning rate 0.05; l2_leaf_reg 3; Bayesian bootstrap; bagging_temperature 1; border_count 64; one_hot_max_size 2; Logloss; AUC evaluation metric; no class weighting. |

**Notes:** Hyperparameters were tuned separately within each outer-training set using inner 5-fold cross-validation. The final harmonized evaluation used five repeats of 5-fold outer cross-validation (25 outer validation splits), with the same predetermined outer partitions applied across models. Consequently, selected hyperparameters varied across outer folds and should not be interpreted as a single globally optimized parameter set. For CatBoost, inner tuning used the first leakage-free imputed dataset within each inner fold, whereas outer validation predictions were averaged across 10 imputations.

**Software environment:** R 4.6.0; mice 3.19.0; pROC 1.19.0.1; glmnet 5.0; randomForest 4.7.1.2; e1071 1.7.17; xgboost 3.2.1.1; catboost 1.2.10.

**Abbreviations:** AUC, area under the receiver operating characteristic curve; IQR, interquartile range; LASSO, least absolute shrinkage and selection operator; SVM, support vector machine.
