# Supplementary material for: Association of NT-proBNP/LVEF with angiographic coronary disease burden in acute myocardial infarction: a retrospective study with machine learning analyses
Source: Front Cardiovasc Med. 2026 Oct 8;13:1964997. doi: 10.3389/fcvm.2026.1964997 (PMC13687254; doi:10.3389/fcvm.2026.1964997)
Supplement: Supplementary file 2 [file Table2.docx]

**Supplementary Table S2. Complete-case sensitivity analysis of model performance**

| **Model** | **Primary analysis AUC (95% CI)** | **Complete-case AUC (95% CI)** | **ΔAUC** | **PR-AUC** | **Brier score** |
| --- | --- | --- | --- | --- | --- |
| Logistic regression | 0.753 (0.704–0.802) | 0.764 (0.706–0.821) | +0.011 | 0.806 | 0.198 |
| LASSO logistic regression | 0.751 (0.702–0.800) | 0.769 (0.711–0.827) | +0.018 | 0.791 | 0.199 |
| Random forest | 0.733 (0.682–0.784) | 0.737 (0.676–0.799) | +0.004 | 0.752 | 0.206 |
| Support vector machine | 0.763 (0.715–0.811) | 0.762 (0.704–0.820) | −0.001 | 0.798 | 0.197 |
| XGBoost | 0.751 (0.702–0.801) | 0.737 (0.675–0.798) | −0.015 | 0.768 | 0.212 |
| CatBoost | 0.740 (0.690–0.790) | 0.737 (0.675–0.798) | −0.003 | 0.759 | 0.209 |

Complete-case analysis included 259 patients with no missing values across the 13 prespecified predictors; 145 (56.0%) had high-burden coronary anatomy. The same repeated 5-fold cross-validation framework with five repeats was used as in the primary analysis, but no multiple imputation was applied. ΔAUC was calculated as complete-case AUC minus primary-analysis AUC. PR-AUC and Brier score are reported for the complete-case analysis.

Abbreviations: AUC, area under the receiver operating characteristic curve; CI, confidence interval; LASSO, least absolute shrinkage and selection operator; PR-AUC, area under the precision-recall curve.
